# Supplementary material for: Microsatellite Markers: A Tool to Assess the Genetic Diversity of Yellow Mustard (Sinapis alba L.)
Source: Plants (Basel). 2023 Nov 29;12(23):4026. doi: 10.3390/plants12234026 (PMC10708087; doi:10.3390/plants12234026)
Supplement: Supplementary file 1 [file plants-12-04026-s001.zip › plants-2698752-supplementary.pdf]

plants

Supplementary Data—Detailed List of Analysed Genetic Resources with Ecn Numbers, Type of Genetic Resource, Country of Origin, Year of Collection and Outputs of STRUCTURE Software.

Table S1. List of analysed genetic resources of yellow mustard and outputs of the STRUCTURE software (Q1/Q2 values).

ID	Name of genetic resource	ECN	Type of genetic resource	Country of origin	Year of collection	Q1 value	Q2 value
1	Bulharská	15O0500001	L	BLG	1985	0.036	0.964
2	Česká krajová	15O0500002	L	CSK	1988	0.026	0.974
3	Dánská 359	15O0500003	L	DEN	1988	0.978	0.022
4	Erbachshofská	15O0500004	L	GER	1988	0.98	0.02
5	Gisilba	15O0500005	V	GER	1957	0.023	0.977
6	Italská	15O0500006	L	-	1988	0.989	0.011
7	Kastor_0	15O0500007	V	-	1988	0.989	0.011
8	Mansholtova	15O0500008	L	GER	1988	0.113	0.887
9	Marocka	15O0500009	L	-	1998	0.982	0.018
10	Moreau Tourne	15O0500010	V	FRA	1988	0.964	0.036
11	Paliisse	15O0500011	V	FRA	1988	0.991	0.009
12	Přerovská bílá	15O0500012	V	CSK	1940	0.021	0.979
13	Rumunia	15O0500013	L	RUM	1988	0.396	0.604
14	Ruska	15O0500014	L	SSSR	1988	0.985	0.015
15	Stalingradska	15O0500015	L	SSSR	1988	0.99	0.01
16	Svalöfská	15O0500016	L	SWE	1988	0.989	0.011
17	Borowska	15O0500018	V	POL	1958	0.027	0.973
18	Dr. Francks	15O0500020	V	GER	1955	0.982	0.018
19	Trico	15O0500021	V	SWE	1973	0.99	0.01
20	Dr. Schneider	15O0500022	V	GER	1973	0.935	0.065
21	K 10	15O0500023	B	DEN	1977	0.984	0.016
22	Albatros	15O0500024	V	GER	1977	0.027	0.973
23	Arde	15O0500025	V	GER	1981	0.972	0.028
24	Carine	15O0500026	V	FRA	1975	0.966	0.034
25	Signal	15O0500028	V	GER	1980	0.013	0.987
26	Budakalaszi Sarga	15O0500030	V	HUN	1972	0.928	0.072
27	Mansholt SIN 13/62	15O0500031	L	GER	1986	0.991	0.009
28	Valdoria	15O0500032	V	GER	1986	0.97	0.03
29	Emergo_0	15O0500033	V	NED	1986	0.329	0.671
30	Siko	15O0500034	V	GER	1986	0.045	0.955
31	Mirly_0	15O0500035	V	AUT	1986	0.989	0.011
32	Maxi_0	15O0500036	V	GER	1986	0.985	0.015
33	Sabre	15O0500037	V	CAN	1987	0.038	0.962
34	Tilney	15O0500038	V	UK	1979	0.985	0.015
35	Mustang	15O0500039	V	SWE	1989	0.045	0.955
36	Perine	15O0500040	V	FRA	1989	0.971	0.029
37	Valiant	15O0500041	V	DEN	1992	0.985	0.015
38	Carnella	15O0500042	V	AUT	1993	0.515	0.485
39	Carnaval	15O0500043	V	FRA	1993	0.907	0.093
40	Fighter	15O0500044	V	FRA	1996	0.983	0.017
41	Nitron	15O0500045	V	DEN	1996	0.956	0.044
42	Braco_0	15O0500046	V	DEN	1997	0.989	0.011
43	Litember	15O0500047	V	GER	1997	0.953	0.047

44	K 395 l	15O0500048	B	SSSR	1997	0.146	0.854
45	Silenda	15O0500049	V	GER	1997	0.02	0.98
46	Martigena	15O0500050	V	GER	1997	0.089	0.911
47	Salvo	15O0500051	V	NED	1997	0.872	0.128
48	Ascot	15O0500052	V	GER	1997	0.015	0.985
49	Metex	15O0500053	V	GER	1995	0.012	0.988
50	K 4116	15O0500054	B	GER	1997	0.96	0.04
51	SIN 10/79_0	15O0500055	B	GER	1996	0.989	0.011
52	KN 29748	15O0500056	B	BLG	1996	0.971	0.029
53	Kramodar	15O0500060	V	SSSR	1963	0.968	0.032
54	Kirby	15O0500061	V	UK	1979	0.989	0.011
55	PGR 0019700	15O0500062	B	CAN	1997	0.906	0.094
56	Comercial Yellow	15O0500063	V	CAN	1997	0.961	0.039
57	Ochre	15O0500064	V	GER	1997	0.985	0.015
58	Local	15O0500065	V	CAN	1997	0.27	0.73
59	Perovska	15O0500066	V	GER	1997	0.021	0.979
60	BGRC 18100	15O0500067	B	GER	1997	0.921	0.079
61	BGRC 28201	15O0500068	B	GREECE	1997	0.983	0.017
62	BGRC 30331	15O0500071	B	GREECE	1997	0.98	0.02
63	BGRC 30330	15O0500072	B	GER	1997	0.036	0.964
64	CGN 13930	15O0500073	B	GER	1991	0.013	0.987
65	Emergo_1	15O0500074	V	NED	1997	0.017	0.983
66	Subotka	15O0500075	V	GER	1998	0.941	0.059
67	Slanski	15O0500076	V	GER	1998	0.21	0.79
68	BGRC 34547	15O0500077	B	GER	1998	0.977	0.023
69	Dr. Schneider	15O0500080	V	GER	1998	0.091	0.909
70	BGRC 34553	15O0500082	B	GER	1998	0.982	0.018
71	VNIIMK	15O0500083	B	GER	1998	0.989	0.011
72	BGRC 34548	15O0500084	B	GER	1998	0.986	0.014
73	Dr. Von Schnieder´s Steinarch	15O0500085	V	GER	1955	0.251	0.749
74	Kastor_1	15O0500086	V	GER	1998	0.17	0.83
75	KN 29737	15O0500087	B	BLG	1998	0.011	0.989
76	Erica	15O0500088	V	FRA	1999	0.564	0.436
77	BGRC 34552	15O0500090	B	SSSR	2000	0.988	0.012
78	BGRC 34555	15O0500091	B	GER	2000	0.987	0.013
79	BRSCHW 34562	15O0500092	B	ROM	2001	0.987	0.013
80	BRSCHW 34563	15O0500093	B	BEL	2001	0.973	0.027
81	BRSCHW 34589	15O0500095	B	GER	2001	0.759	0.241
82	BRSCHW 55705	15O0500097	B	AUS	2001	0.028	0.972
83	Tilny	15O0500098	V	GER	2001	0.683	0.317
84	Severka_0	15O0500099	V	CZE	2003	0.014	0.986
85	Medicus	15O0500100	V	SWE	2006	0.024	0.976
86	YE-278	15O0500103	B	Izrael	2008	0.05	0.95
87	SIN 10/79_1	15O0500105	B	-	2009	0.982	0.018
88	BRGC 34548	15O0500106	B	-	2009	0.988	0.012
89	Seco	15O0500107	V	GER	2009	0.954	0.046
90	Semper	15O0500108	V	GER	2009	0.925	0.075
91	Setoria	15O0500109	V	GER	2009	0.955	0.045
92	Signo	15O0500110	V	GER	2009	0.023	0.977
93	Sigri	15O0500111	V	GER	2009	0.988	0.012

94	Simona	15O0500112	V	GER	2009	0.956	0.044
95	Sirte	15O0500113	V	GER	2009	0.973	0.027
96	Sunshine_0	15O0500114	V	SWE	2009	0.01	0.99
97	Sito	15O0500116	V	GER	2010	0.027	0.973
98	Solea	15O0500117	V	BEL	2010	0.019	0.981
99	Salsa_0	15O0500118	V	BEL	2010	0.089	0.911
100	Polka_0	15O0500119	V	BEL	2010	0.034	0.966
101	ABA	15O0500120	V	NED	2015	0.025	0.975
102	King	15O0500121	V	GER	2013	0.028	0.972
103	Amog	15O0500122	V	NED	2015	0.037	0.963
104	Chacha	15O0500123	V	BEL	2008	0.107	0.893
105	Ultra	15O0500124	V	NED	2016	0.011	0.989
106	Admiral	15O0500125	V	NED	2016	0.01	0.99
107	Andromeda_0	15O0500126	V	CZE	2014	0.016	0.984
108	47-635-YE 13	15O05	B	-	-	0.033	0.967
109	Abraham	15O05	V	-	-	0.953	0.047
110	AC Base	15O05	V	-	-	0.171	0.829
111	Agent_0	15O05	V	CZE	2016	0.974	0.026
112	Achilles	15O05	V	-	-	0.93	0.07
113	Architect	15O05	V	-	-	0.937	0.063
114	Atlet	15O05	V	-	-	0.987	0.013
115	Attack	15O05	V	-	-	0.984	0.016
116	Bonus	15O05	V	-	-	0.824	0.176
117	Brisant	15O05	V	-	-	0.012	0.988
118	Concerta	15O05	V	-	-	0.086	0.914
119	Esprit	15O05	V	-	-	0.076	0.924
120	Flenal	15O05	V	-	-	0.152	0.848
121	Iris	15O05	V	-	-	0.075	0.925
122	Maxi_1	15O05	V	GER	1986	0.054	0.946
123	Mega	15O05	V	GER	2011	0.067	0.933
124	Merigue	15O05	V	-	-	0.134	0.866
125	Mikado	15O05	V	-	-	0.015	0.985
126	Octopus	15O05	V	-	-	0.009	0.991
127	Passion	15O05	V	-	-	0.011	0.989
128	Profi	15O05	V	-	-	0.023	0.977
129	Protect	15O05	V	GER	2016	0.034	0.966
130	Sabon	15O05	V	-	-	0.095	0.905
131	Samba_0	15O05	V	-	-	0.016	0.984
132	Santa Fé	15O05	V	-	-	0.052	0.948
133	Sigma	15O05	V	-	-	0.041	0.959
134	Sirtaky	15O05	V	-	-	0.016	0.984
135	Swing	15O05	V	BEL	2016	0.025	0.975
136	Thorney	15O05	V	CAN	2008	0.056	0.944
137	Torpedo	15O05	V	-	-	0.009	0.991
138	Venice	15O05	V	-	-	0.02	0.98
139	Veto_1	15O05	V	GER	2015	0.025	0.975
140	Viscount	15O05	V	CAN	2008	0.054	0.946
141	Vitaro	15O05	V	-	-	0.021	0.979
142	Warta	15O05	V	-	-	0.034	0.966
143	Elendil	15O05	V	-	-	0.03	0.97
144	Condor	15O05	V	CAN	2008	0.951	0.049

145	Maxi_2	15O05	V	GER	2015	0.026	0.974
146	Andromeda_1	15O0500126	V	CZE	2014	0.042	0.958
147	Sunshine_1	15O0500114	V	SWE	2009	0.035	0.965
148	Veto_2	15O0500127	V	GER	2015	0.015	0.985
149	Agent_1	15O0500132	V	CZE	2016	0.95	0.05
150	Veronika	15O0500115	V	CZE	2010	0.968	0.032
151	Polka_1	15O0500119	V	BEL	2010	0.02	0.98
152	Polarka	15O0500101	V	CZE	2006	0.017	0.983
153	Salsa_1	15O0500118	V	BEL	2010	0.018	0.982
154	Zlata	15O0500027	V	CSK	1983	0.014	0.986
155	Severka_1	15O0500099	V	CZE	2003	0.045	0.955
156	Samba_1	15O05	V	-	-	0.016	0.984
157	Otava (OP-SA-01)		V	CZE	2018	0.024	0.976
158	Olga (OP-SA-02)		V	CZE	2018	0.032	0.968
159	OP-2282		B	CZE	2018	0.038	0.962
160	OP-2288		B	CZE	2018	0.028	0.972
161	OP-2291		B	CZE	2018	0.023	0.977
162	OP-2379		B	CZE	2018	0.035	0.965
163	OP-2408		B	CZE	2018	0.03	0.97
164	OP-2415		B	CZE	2018	0.026	0.974
165	OP-2443		B	CZE	2018	0.044	0.956
166	OP-2450		B	CZE	2018	0.042	0.958
167	Asta	15O05	V	-	-	0.019	0.981
168	Fox	15O05	V	-	-	0.011	0.989
169	Rumba	15O05	V	-	-	0.008	0.992
170	Serval	15O05	V	-	-	0.017	0.983
171	Mirly_1	15O05	V	AUT	1986	0.022	0.978
172	Sinus	15O05	V	-	-	0.008	0.992
173	Ludique	15O05	V	-	-	0.021	0.979
174	Bamberka	15O05	V	-	-	0.951	0.049
175	Budakalászi sárga	15O05	V	HUN	-	0.023	0.977
176	Braco_1	15O05	V	DEN	1997	0.014	0.986
177	Raduga	15O05	V	-	-	0.022	0.978
178	Panter	15O05	V	-	-	0.013	0.987
179	Smash	15O05	V	-	-	0.009	0.991
180	Cratos	15O05	V	-	-	0.009	0.991
181	Ultimo	15O05	V	-	-	0.011	0.989
182	Silvester	15O05	V	-	-	0.009	0.991
183	Sibelius	15O05	V	-	-	0.008	0.992
184	Symbol	15O05	V	-	-	0.01	0.99
185	Budakalaszi sárga	15O0500030	V	HUN	1986	0.014	0.986
186	Olga	15O05	V	CZE	2018	0.007	0.993
187	Otava	15O05	V	CZE	2018	0.013	0.987
188	TIL_6824_1		BT	CZE		0.006	0.994
189	TIL_6824_2		BT	CZE	2022	0.012	0.988
190	TIL_6824_3		BT	CZE	2022	0.01	0.99
191	TIL_6824_4		BT	CZE	2022	0.008	0.992
192	TIL_6824_5		BT	CZE	2022	0.008	0.992
193	TIL_6824_6		BT	CZE	2022	0.027	0.973
194	TIL_6824_7		BT	CZE	2022	0.009	0.991
195	TIL_6824_8		BT	CZE	2022	0.012	0.988

196	TIL_6824_9	BT	CZE	2022	0.007	0.993
197	TIL_6824_10	BT	CZE	2022	0.01	0.99
198	TIL_6824_11	BT	CZE	2022	0.008	0.992

ID = identification number, L – landrace, V – variety, B – breeding material, BT – breeding material from TILLING population

Table S2. Detailed output of cluster number estimation and statistics for different values of K.

K	Reps	Mean LnP(K)	Stdev LnP(K)	Ln′(K)	ǀLn''(K)ǀ	Delta K
1	25	-2827.2680	0.0557	NA	NA	NA
**2**	**25**	**-2527.2080**	**0.9742**	**300.060000**	**138.444000**	**142.107877**
3	25	-2365.5920	18.8927	161.616000	101.932000	5.395312
4	25	-2305.9080	60.4267	59.684000	9.364000	0.154965
5	20	-2236.8600	16.2001	69.048000	25.453000	1.571165
6	20	-2193.2650	34.4489	43.595000	77.130000	2.238970
7	20	-2226.8000	155.0820	-33.535000	71.080000	0.458338
8	20	-2189.2550	144.2788	37.545000	NA	NA

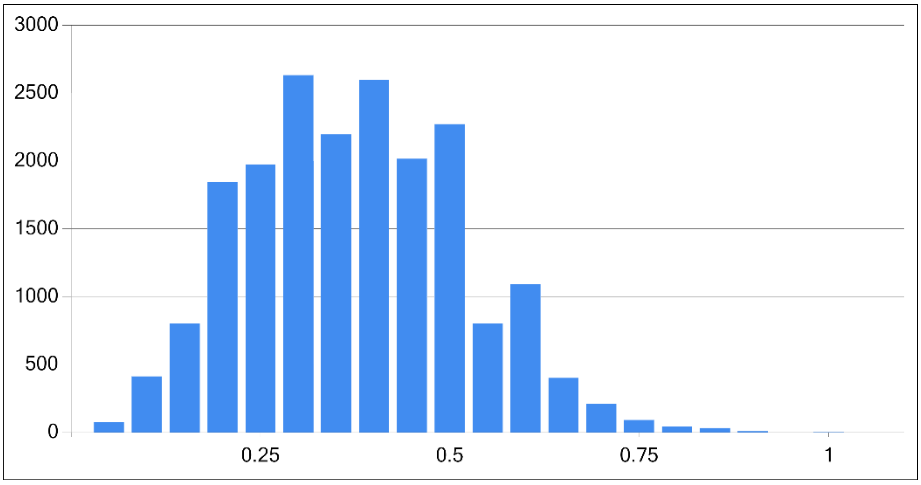

Figure S1. The graphic representation of the degree of dissimilarity between genetic resources - output from DARwin 6.0 software.
